# 

**Published:** 1880-11

**Authors:** 


### THE OHIO STATE DENTAL SOCIETY.

The following list of subjects are presented for consideration, at the meeting of the Ohio State Dental Society, to be held in Columbus on the first Tuesday of December, 1880:

1st. Pathology of the osseous structure of the teeth.

2d. Popular dental education.

3d. Preparation of cavities for gold filling.

4th. Mechanical Dentistry—Partial sets of artificial teeth, secured by clasps, also by atmospheric pressure, which is the preferable method?

5th. Oral surgery.

6th. Professional courtesies.

7th. Treatment of inflammation of the dental periosteum.

W. W. Sillito, Rec. Sec.

### STATE MEETING.

The fifteenth annual meeting of the Ohio State Dental Society will be held in Columbus, on Tuesday, the 7th of December next.

As will be seen on another page of this Number, an interesting and inviting programme, will be presented for discussion.

It is desirable that every member of the Society be present and bring at least one good man with him, who may become a member, or at least be an interested visitor. The society should have a membership of two hundred and fifty to three hundred, and these should all be active workers in the body. Every one should be prepared to do *something* for the general good; each one should study well the subjects that are proposed for the occasion and be ready to give his views and opinions upon each. Such preparation will do the individual himself good, and the presentation of his thoughts and opinions will do others good. Let there be a goodly number of well digested papers, and in addition every one should be prepared with mature thought for discussion. All interesting and instructive cases should be brought forward; all new or improved instruments and appliances should be exhibited.

It is very unfortunate that so many in our societies, do little or nothing except to be present and enjoy the meetings; this, of course is better than to be absent, but it falls far short of accomplishing the greatest good. If every one would do what he could in society work, the advancement and elevation of the profession would be greatly enhanced.

There are subjects of general interest that should always be considered, especially at State meetings, viz: Professional education, the legal status of the profession, the ethics of the profession.

The programme of the meeting is doubtless in the hands of the members ere this. Let it be well studied, and may the meeting of 1880 be larger than ever before.

### EXAMINING BOARD.

The annual meeting of the "Board of Dental Examiners" will be held in the city of Columbus, on Wednesday, December 8th, at 12 o'clock. All interested will facilitate business by being present at that time as promptly as possible.

F. H. Rehwinkel, Sec.

### REVIVAL.

The Cincinnati Dental society, which has been resting for three or four years, has aroused itself and concluded to go to work. A meeting has been called, to be held on Tuesday evening, at half past seven o'clock, November 23, 1880, in the lecture room of the Ohio College of Dental Surgery. It is very desirable that all the members be present, and bring their professional friends with them. Let all come prepared to make it an interesting occasion.

A MONTHLY MAGAZINE DEVOTED TO THE INTERESTS OF

**THE DENTAL PROFESSION.**

*TERMS REDUCED TO $2.50 PER YEAR. SINGLE COPIES 25C.*

EDITED BY PROF. J. TAFT, D.D.S.

AND

*Published by SPENCER, CROCKER & CO., Ohio Dental Depot.*

The volume commences in January and closes in December.

The REGISTER being issued monthly is always fresh, therefore Indispensable to the practitioner who desires to keep posted up with the new inventions and improvements which are daily developed. Neither expense nor effort will be spared to give value and variety to its contents. Articles will be illustrated with well executed engravings whenever they will add to the interest or assist to explanation.

Its extensive circulation and frequency of issue make it one of the BEST DENTAL ADVERTISING MEDIUMS in the United States.

All subscriptions must begin with January or July.

Local or special notices, five lines or less, will be inserted for$3 each insertion; over five lines, 50 cts. per line.

All advertising bills payable in advance, or at furthest, after the issue of the first number containing the advertisement. All transient advertisements to be paid for in advance.

☞CONTRIBUTIONS SOLICITED.

Exclusively Editorial Communications should be addressed to Editor.

The latest number of the REGISTER may be ordered with or without other goods at any time, and all letters should be addressed to

*SPENCER, CROCKER & CO., Ohio Dental Depot, Cincinnati, O.*

